# Hemophagocytic Lymphohistiocytosis Thought to Be due to Undifferentiated Connective Tissue Disease Treated With Ruxolitinib: A Case Report

**DOI:** 10.1155/crrh/9165820

**Published:** 2026-10-18

**Authors:** Emily Vachon, Heena Birbal Jain, Suzanna Kitten

**Affiliations:** ^1^ Carle Illinois College of Medicine Urbana Illinois USA; ^2^ Carle Foundation Hospital Urbana Illinois USA carle.org

**Keywords:** case report, hemophagocytic lymphohistiocytosis, ruxolitinib, undifferentiated connective tissue disease

## Abstract

Hemophagocytic lymphohistiocytosis (HLH) is a life‐threatening, systemic inflammatory syndrome. Cases without an identifiable acquired trigger are referred to as secondary HLH. In this report, we describe the case of a 24 year‐old woman whose underlying cause of HLH was particularly difficult to determine. Her condition was initially suspected to be due to lamotrigine use but is now thought to be a result of undifferentiated connective tissue disease. During the patient’s prolonged and complex hospital stay, she received the diagnosis of secondary HLH. Discontinuing lamotrigine, a drug that rarely causes HLH, did not improve her condition. The patient was tested for infectious and malignant conditions with negative results. Further workup revealed a positive ANA and bilateral MCP tenosynovitis, with negative specific antibodies, suggesting an undefined rheumatologic process. A number of conventional therapies did not show clear benefit, including etoposide, high‐dose steroids, rituximab, and plasmapheresis. The patient remained in critical condition until she began treatment with ruxolitinib, with which she remains stable more than 5 years later. Ruxolitinib has shown some success in treating HLH in preliminary studies, but it is still considered a salvage therapy. This case illustrates the successful use of ruxolitinib in a patient with HLH likely due to undifferentiated connective tissue disease.

## 1. Introduction

Hemophagocytic lymphohistiocytosis (HLH) is a life‐threatening, systemic inflammatory syndrome characterized by pathologic activation of T cells and excessive production of interferon‐γ (IFN‐γ), which promotes activation and accumulation of macrophages in tissues and contributes to hypercytokinemia and multiorgan failure. The autoimmune dysregulation in HLH leads to hypercytokinemia and accumulation of activated macrophages in organs, often resulting in multiorgan failure. HLH is classified as either primary or secondary, where primary cases are due to genetic mutations and secondary cases are due to an acute illness trigger. Triggers of secondary HLH include infection, malignancy, drugs, and autoimmune disorders. In the case of an autoimmune etiology, HLH is often referred to as macrophage activation syndrome (MAS). Per the HLH‐2004 criteria, the diagnosis of secondary HLH is made if 5 out of 8 quantitative diagnostic criteria are met (Table 1).

**TABLE 1:** HLH‐2004 diagnostic criteria^a^.

1. Fever

2. Splenomegaly

3. Cytopenia in ≥ 2 of the following peripheral blood lineages: a. Hemoglobin < 90 g/L b. Platelets < 100 × 10^9^/L c. Neutrophils < 1.0 × 10^9^/L

4. Hypertriglyceridemia (fasting triglycerides ≥ 265 mg/dL) and/or hypofibrinogenemia (fibrinogen ≤ 1.5 g/L)

5. Evidence of hemophagocytosis in bone marrow, spleen, or lymph nodes, without evidence of malignancy

6. Low or absent NK‐cell activity

7. Ferritin ≥ 500 μg/L

8. Soluble CD25 ≥ 2400 U/mL

*Note:* CD25: Cluster of Differentiation 25.

Abbreviation: NK, natural killer.

^a^Data adapted from Henter et al. [1].

We describe the complex case of a 24‐year‐old female with secondary HLH, originally suspected to be a result of lamotrigine therapy. The patient was eventually treated successfully with ruxolitinib and remains in remission more than 5 years later with continued treatment. Though investigation is still underway, the patient’s HLH is thought to be secondary to undifferentiated connective tissue disease (UCTD).

## 2. Case Presentation

A 24‐year‐old female patient with a history of chronic autoimmune thyroiditis (Hashimoto’s disease) leading to hypothyroidism, obsessive–compulsive disorder, persistent depressive disorder, and posttraumatic stress disorder presented to the emergency department with jaundice, nausea, vomiting, fever, and body aches. Her initial laboratory study revealed a decreased hemoglobin from baseline (9.5 g/dL), elevated total bilirubin (1.5 mg/dL), elevated lactate dehydrogenase (LDH) of 577 U/L, and low haptoglobin (< 8.0 mg/dL). Given these findings, the patient was admitted and started on intravenous (IV) steroid treatment. The patient’s condition rapidly declined and did not appear to be responsive to steroids. Notably, her hemoglobin dropped to 4.7 g/dL in less than 24 h, reaching a nadir of 3.1 g/dL in less than 3 days despite multiple transfusions. Her bilirubin also rose to 17 mg/dL and LDH to 1016 U/L, and a direct Coombs test displayed positive warm autoantibodies. The decision was made to transfer the patient to a hospital with a higher level of care.

Upon transfer, the patient underwent a bone marrow biopsy, which revealed moderate hemophagocytosis, but no evidence of leukemia or lymphoma was found. She was diagnosed with HLH based on the biopsy results, pancytopenia, hyperferritinemia (peak of 58,505 μg/L), splenomegaly per abdominal ultrasound, hypertriglyceridemia (269 mg/dL), ad elevated and high fevers. Soluble CD25 was tested but only mildly elevated at 1385 U/mL. Natural killer cell activity results were unable to be located in the chart. Therefore, the patient met six out of eight HLH‐2004 criteria. A CXCL9 level was not identified in the available medical record. At this point, multiple specialties began investigating the underlying cause. Full‐body positron‐emission tomography/computed tomography (PET/CT) was negative for lymphoma, and skin biopsy was negative for intravascular lymphoma. Infectious disease workup, including cytomegalovirus (CMV), Epstein–Barr virus (EBV), parvovirus, *Ehrlichia*, respiratory virus panel, and blood cultures, was also unrevealing.

In addition to investigating potential triggers of secondary HLH, primary HLH was also considered. Primary HLH has historically been considered a disease of infants and young children, but recent studies with more robust genetic testing have called this into question. In a 2011 study of 1531 HLH patients by Zhang et al., 12% of patients were diagnosed with HLH at the age of 18 years or older [2]. Genetic testing was negative for mutations in the four primary HLH‐associated genes: PRF1, STX11, STXBP2, and UNC13D. The patient was also negative for mutations in genes encoding for other immunodeficiency syndromes and inborn errors of metabolism that can present with HLH phenotypes (Table 2). Additionally, the patient’s NK cell perforin mean channel fluorescence (MCF) was elevated above the reference range of 98–181. Low or absent perforin MCF is associated with RF1‐related HLH (familial HLH Type 2), but perforin expression may be normal or elevated in primary HLH caused by defects in cytotoxic lymphocyte degranulation, including variants in STX11, STXBP2, and UNC13D [5]. Thus, the patient’s elevated perforin MCF does not exclude primary HLH due to degranulation defects, although the absence of pathogenic variants on genetic testing makes these disorders less likely. A CD107a degranulation assay was not performed, which would have provided additional functional assessment for defects in cytotoxic lymphocyte degranulation.

**TABLE 2:** Nonfamilial HLH genes included in genetic testing^a^.

Gene	Associated disorder
AP3B1	Hermansky–Pudlak syndrome
BLOC1S6‐PLDN	Hermansky–Pudlak syndrome
GATA2	GATA2 deficiency syndrome
ITK	Lymphoproliferative syndrome 1
LYST	Chediak–Higashi syndrome
MAGT1	Immunodeficiency, XL, w/magnesium defect, EBV infection and neoplasia
RAB27A	Griscelli syndrome Type 2
SH2D1A	X‐linked lymphoproliferative disease 1
SLC7A7	Lysinuric protein intolerance
CD27	Lymphoproliferative syndrome 2
XIAP	X‐linked lymphoproliferative disease 2

^a^Data adapted from Zhang et al. [3] and Wu et al. [4].

The patient was evaluated by rheumatology for underlying autoimmune etiology of her HLH. The only notable rheumatologic symptom the patient reported prior to admission was bilateral stiffness in her first two metacarpophalangeal (MCP) and proximal interphalangeal (PIP) joints. She saw her primary care provider earlier in the year and was found to have negative rheumatoid factor (RF), cyclic citrullinated peptide (CCP), antinuclear antibody (ANA), and hand radiographs. During her initial admission, prior to transfer, ANA was again negative. However, after transfer, repeat ANA testing was positive at a titer of 1:160 with a homogeneous pattern, increasing to 1:320 with a speckled pattern on repeat testing the following day. A summary of rheumatologic laboratory results checked upon transfer are summarized in Table 3. Magnetic resonance imaging (MRI) revealed bilateral second MCP synovitis, but other serologic testing was unrevealing. Given the presence of inflammatory arthritis and a persistently positive ANA without sufficient clinical or serologic evidence to meet criteria for a defined connective tissue disease, the patient was diagnosed with UCTD.

**TABLE 3:** Autoimmune laboratory results.

Test	Result	Reference range
Rheumatoid factor	< 10.0 units/mL	0.1–15 units/mL
Anti‐CCP	< 0.5 units/mL	< 3 units/mL
Anti‐dsDNA	2.0 units/mL	< 4 units/mL
ANA (day 1)	1:160, homogeneous	< 1:80
ANA (day 2)	1:320, speckled	< 1:80
ENA antibody panel (Jo‐1, RNP, Svl‐70, Smith, SS‐A/Ro, and SS‐B/La antibodies)	Negative	Negative
Lupus anticoagulant	Negative	Negative
Anticardiolipin antibodies (IgG)	< 1.6 U/mL	< 20 U/mL
Anticardiolipin antibodies (IgM)	< 0.2 U/mL	< 20 U/mL
Anti‐β2‐glycoprotein I (IgG)	< 1.4 U/mL	< 20 U/mL
Anti‐β2‐glycoprotein I (IgM)	< 0.2 U/mL	< 20 U/mL

Another leading theory involved the patient’s lamotrigine use. The patient began taking lamotrigine 2 years before presenting to the emergency department with HLH symptoms. This medication was added to address the patient’s persistent depressive symptoms with mixed features after an inadequate response to fluvoxamine. Her dose was increased from 100 to 150 mg twice daily about 8 months prior to admission. The medication was fairly effective for her but was stopped immediately upon diagnosis of HLH. Lamotrigine is known to trigger immune‐related adverse reactions, including HLH, though this is exceedingly rare [6].

Following transfer to the second hospital, the patient was treated with multiple immunomodulatory therapies. She received two doses of rituximab, two rounds of plasmapheresis, and one dose of intravenous immunoglobulin (IVIG). The HLH‐2004 protocol was initiated with dexamethasone 10 mg/m^2^ daily and etoposide 75 mg/m^2^. One dose of etoposide was administered prior to initiation of ruxolitinib, with a second dose administered one week later. Ruxolitinib was initiated at 5 mg twice daily (BID) and gradually increased to 20 mg BID. Alemtuzumab 0.25 mg/kg daily was subsequently administered for 4 days while ruxolitinib was ongoing. Within several days of initiating ruxolitinib, her hemoglobin began to rise, and her C‐reactive protein (CRP) and ferritin levels began to decrease. Within a couple of weeks, the patient’s laboratory abnormalities had nearly normalized, and she was discharged on prednisone 30 mg BID and ruxolitinib 20 mg BID.

The patient remained stable after discharge, and after a few months, her hematology team made the decision to begin tapering off ruxolitinib. She stopped completely a year after discharge. Two months later, she presented to the emergencydepartment with nausea, vomiting, and abdominal pain. Her hemoglobin dropped to 7.3 g/dL, down from her baseline of about 13 g/dL. Her ferritin was elevated at 2798 ng/mL, and her CRP was elevated at 18.8. She was admitted and found to have a splenic infarction. A bone marrow biopsy again showed hemophagocytic histiocytes. This was consistent with a flare of her HLH, and ruxolitinib 20 mg BID was restarted. Her inflammatory laboratory results quickly improved, and she was discharged.

About 7 years after initial hospitalization, the patient’s oncology team began another gradual taper of ruxolitinib due to her stable condition and possible side effects. Once her dose was reduced to 5 mg BID, she again experienced an HLH flare, albeit milder than in the past. On presentation to the emergency department, the patient’s hemoglobin was 8.2 g/dL, and ferritin was 1340 ng/mL. CRP was not checked. Given this milder presentation, the patient was comfortable with increasing ruxolitinib back to 20 mg and monitoring her symptoms at home. Her blood counts improved significantly with this increased dose, and she eventually returned to baseline. At the time of writing, she remains stable on 20 mg ruxolitinib BID.

## 3. Discussion

Management of secondary HLH in adults can be particularly challenging when an underlying trigger cannot be definitively identified. In this case, extensive evaluation for genetic, infectious, malignant, and rheumatologic causes did not identify a clear etiology. Although the patient was initially thought to have lamotrigine‐associated HLH, the timing of symptom onset relative to initiation and dose escalation of lamotrigine made this explanation less likely. A 2019 review of the Food and Drug Administration’s Adverse Event Reporting System found 8 cases of lamotrigine‐associated HLH between 1994 and 2017 [6]. However, in 7 out of 8 of these, cessation of the drug resolved the condition. Additionally, all cases presented less than 24 days after beginning lamotrigine. In this case, her symptoms did not start until 8 months after her last dose increase and about 2 years after starting the medication.

The patient’s subsequent diagnosis of UCTD provides a possible explanation for her HLH, although a definitive causal relationship cannot be established. Systemic lupus erythematosus and systemic juvenile arthritis are the two most common rheumatologic causes of HLH [7]. However, sporadic cases of HLH secondary to UCTD have been reported in the literature [8]. In rare cases, HLH can be the first presentation of an autoimmune disorder, even years before the autoimmune condition manifests [9]. Thus, an underlying rheumatologic disorder remains a possible driver of HLH in this patient, although the absence of a defined autoimmune diagnosis highlights the challenge of treating HLH when the underlying trigger remains uncertain.

The most notable therapeutic aspect of this case was the sustained response associated with ruxolitinib. Ruxolitinib is a Janus kinase (JAK) 1/2 inhibitor approved for use in myeloproliferative neoplasms and steroid‐resistant graft‐versus‐host disease. Ruxolitinib’s JAK inhibition results in modulation of multiple inflammatory factors implicated in HLH, including IL‐2, IL‐6, IL‐8, IL‐10, IL‐12, IL‐18, TNF, and IFN‐γ [10]. Increasing evidence supports the use of ruxolitinib in adults with secondary HLH, particularly in refractory or treatment‐resistant disease [10–12]. In a recent retrospective cohort, clinical benefit was numerically more frequent among patients with rheumatic HLH than among those with nonrheumatic HLH (86% vs 53%); however, this difference was not statistically significant, and survival benefit among responders was also observed in nonrheumatic HLH [10]. The clinical course of this case adds to the evidence supporting the use of ruxolitinib as a salvage therapy in refractory secondary HLH. In patients with secondary HLH in whom an underlying trigger remains uncertain, treatment decisions must balance the need for rapid control of hyperinflammation with the toxicity and uncertain efficacy of available therapies. Although etoposide and alemtuzumab were used in this patient, the subsequent sustained response to ruxolitinib raises the possibility that JAK inhibition may represent an alternative therapeutic option in selected patients with refractory or undifferentiated HLH. However, the available evidence is insufficient to recommend ruxolitinib over established HLH‐directed therapies or to define its optimal position in the treatment sequence.

Of note, ruxolitinib was initiated shortly after the first dose of etoposide, and alemtuzumab was also administered concurrently with ruxolitinib. Therefore, the initial clinical improvement cannot be attributed to ruxolitinib alone. However, the subsequent clinical course provides additional support for a therapeutic role of ruxolitinib. The treatment team attempted to discontinue maintenance ruxolitinib on two separate occasions, and each attempt was followed by an HLH flare that improved after ruxolitinib was resumed. This repeated association between ruxolitinib discontinuation and disease relapse supports a role for ruxolitinib in maintaining remission.

## Funding

This work received no specific grant from any funding agency in the public, commercial, or not‐for‐profit sectors.

## Ethics Statement

Institutional review board approval was not required for this case report in accordance with institutional policy.

## Consent

Written informed consent was obtained from the patient for publication of this case report.

## Conflicts of Interest

The authors declare no conflicts of interest.

## Supporting Information

Additional supporting information can be found online in the Supporting Information section.

## Supporting information


**Supporting Information** HLHCareChecklist.

## Data Availability

Data sharing is not applicable to this article as no new data were created or analyzed in this study.
