# Supplementary material for: Hemophagocytic Lymphohistiocytosis Thought to Be due to Undifferentiated Connective Tissue Disease Treated With Ruxolitinib: A Case Report
Source: Case Rep Rheumatol. 2026 Oct 18;2026:9165820. doi: 10.1155/crrh/9165820 (PMC13677135; doi:10.1155/crrh/9165820)
Supplement: Supplementary file 1 [file CRRH-2026-9165820-s001.docx]

Hemophagocytic Lymphohistiocytosis Thought to Be Due to Undifferentiated Connective Tissue Disease Treated With Ruxolitinib: A Case Report

Emily Vachon, BA (vachon2@illinois.edu), Heena Birbal Jain, MD (heena.birbaljain@carle.com), Suzanna Kitten, MD ([suzanna.kitten@carle.com](mailto:suzanna.kitten@carle.com))

Care Checklist:

1. Title – The diagnosis or intervention of primary focus followed by the words “case report” - **Complete**
2. Key Words – 2 to 5 key words that identify diagnoses or interventions in this case report (including "case report") - **Complete**
3. Abstract – **Complete**
   - Introduction – What is unique about this case and what does it add to the scientific literature?
   - The patient’s main concerns and important clinical findings.
   - The primary diagnoses, interventions, and outcomes.
   - Conclusion – What are one or more “take-away” lessons from this case report?
4. Introduction – Briefly summarizes why this case is unique and may include medical literature references. - **Complete**
5. Patient Information - **Complete**
   - De-identified patient specific information.
   - Primary concerns and symptoms of the patient.
   - Medical, family, and psychosocial history including relevant genetic information.
   - Relevant past interventions and their outcomes.
6. Clinical Findings – Describe significant physical examination (PE) and important clinical findings - **Complete**
7. Timeline – Historical and current information from this episode of care organized as a timeline (figure or table) – **The timeline is currently organized in a narrative format rather than a figure or table. The first draft of the manuscript had a table, but this was noted by the reviewers to be very busy and difficult to follow. The patient’s clinical course is very complex and the authors have opted to remove the timeline figure for this reason. However, the narrative timeline was expanded upon for further clarity.**
8. Diagnostic Assessment - **Complete**
   - Diagnostic methods (PE, laboratory testing, imaging, surveys).
   - Diagnostic challenges.
   - Diagnosis (including other diagnoses considered).
   - Prognostic characteristics when applicable.
9. Therapeutic Intervention - **Complete**
   - Types of therapeutic intervention (pharmacologic, surgical, preventive).
   - Administration of therapeutic intervention (dosage, strength, duration).
   - Changes in therapeutic interventions with explanations.
10. Follow-up and Outcomes - **Complete**
    - Clinician- and patient-assessed outcomes if available.
    - Important follow-up diagnostic and other test results.
    - Intervention adherence and tolerability. (How was this assessed?)
    - Adverse and unanticipated events.
11. Discussion - **Complete**
    - Strengths and limitations in your approach to this case.
    - Discussion of the relevant medical literature.
    - The rationale for your conclusions.
    - The primary “take-away” lessons from this case report (without references) in a one paragraph conclusion.
12. Patient Perspective – The patient should share their perspective on the treatment(s) they received. – **Not included – we do not have this information**
13. Informed Consent – The patient should give informed consent. - **Complete**
